# Clinical characteristics and hearing loss etiology of cochlear implantees undergoing surgery in their teens, 20s, and 30s

**DOI:** 10.1007/s00405-024-08737-3

**Published:** 2024-05-27

**Authors:** Goun Choe, Jong Woo Lim, Ye Jun Chun, Jin Hee Han, Bong Jik Kim, Byung Yoon Choi

**Affiliations:** 1 Department of Otolaryngology-Head and Neck Surgery, Chungnam National University College of Medicine, Chungnam National University Sejong Hospital, Sejong, Republic of Korea; 2 Department of Otorhinolaryngology, Seoul National University Bundang Hospital, Seongnam, Republic of Korea; 3 Sensory Organ Research Institute, Seoul National University Medical Research Center, Seoul, Republic of Korea

**Keywords:** Cochlear implantation, Adolescent, Young adult, Teenager, Speech outcome

## Abstract

**Purpose:**

This study aimed to investigate the etiology of hearing loss, including genetic variants, in individuals who underwent cochlear implantation (CI) in their teens to thirties. It also sought to analyze post-CI speech performance and identify prognostic factors affecting CI outcomes in this age group.

**Methods:**

We conducted a retrospective review of 421 cochlear implant patients at Seoul National University Bundang Hospital, focusing on 63 subjects aged 10–39 years who underwent their first CI by a single surgeon between July 2018 and June 2022. The study included audiologic evaluation, molecular genetic testing, and analysis of speech performance post-CI. Statistical analyses were performed using SPSS 25 and GraphPad Prism 7.

**Results:**

Among 63 participants (M:F, 24:39), nine underwent CI in their teens, 24 in their 20 s, and 30 in their 30 s. Most of them (40, 63.5%) had postlingual deafness. The study found that 65.2% (40/63) of subjects received a genetic diagnosis, with DFNB4 being the most common etiology (37.5%, 15/40). Post-CI speech evaluation showed an average sentence score of 80% across all subjects. Factors such as the onset of hearing loss, duration of deafness (DoD), and preoperative Speech Intelligibility Rating (SIR) significantly influenced CI outcomes. Notably, longer DoD was associated with poorer CI outcomes, but this did not affect individuals with postlingual hearing loss as much.

**Conclusion:**

The study concludes that in individuals aged 10–39 undergoing CI, the onset of hearing loss and preoperative SIR are critical predictors of postoperative outcomes. CI is recommended for those with postlingual hearing loss in this age group, irrespective of the DoD. The study highlights the importance of genetic factors especially DFNB4 in hearing loss etiology and underscores the value of the relatively easy-to-evaluate factor, preoperative SIR in predicting CI outcomes.

**Supplementary Information:**

The online version contains supplementary material available at 10.1007/s00405-024-08737-3.

## Introduction

Cochlear implantation (CI) is recognized as the primary option for rehabilitation of severe to profound hearing loss. Traditionally, CI has been most common in two distinct groups: pre-lingually or peri-lingually deafened pediatric subjects, who often receive CI at a young age, and older adults, whose need for CI is growing in an aging society. This has led to a dichotomized age distribution among cochlear implantees.

However, there exists an underrepresented niche demographic within cochlear implantees: individuals in their teens to thirties. Extensive research has explored the anatomical and genetic causes of hearing loss in pediatric populations, a topic that continues to attract significant attention. Similarly, for older implantees, hearing loss is often attributed to postlingual factors or genes associated with late-onset hearing loss. Previous studies have shown that both groups, when implanted timely, can experience satisfactory auditory rehabilitation.

Despite this knowledge, the causes of hearing loss in individuals seeking CI between their teens and thirties remain less understood, as does their prognosis following CI. This age group has rarely been the focus of research, and when it has, the studies often involve cases of revision surgery or bilateral sequential second surgeries.

In this study, we aimed to comprehensively explore the etiology of hearing loss, encompassing genetic variants, the onset of hearing loss, duration of deafness (DoD), and preoperative speech intelligibility rating (SIR), in individuals who underwent CI in their teens to thirties. Our analysis specifically focused on how these factors influence speech performance post-CI. The patient cohort comprised individuals who received their first CI from a single surgeon at a single center, allowing for a focused and consistent examination of these factors and their impact on postoperative outcomes.

## Materials and methods

### Subjects

We conducted a retrospective review of the medical records of all individuals who underwent cochlear implantation by a single surgeon (C.B.Y) at Seoul National University Bundang Hospital between July 2018 and June 2022, totaling 421 subjects. From this group, we selected subjects who received their first CI between the ages of 10 and 39 years, specifically excluding those who underwent revision or sequential secondary procedures. The onset of hearing loss was categorized into three groups: prelingual, perilingual, and postlingual. Prelingual hearing loss was defined as bilateral severe or greater degree of hearing loss occurring before the age of 4 years [1, 2]. Hearing loss occurring between the ages of 4 and 6 years was classified as perilingual deafness, and deafness occurring after that period was considered postlingual.

We reviewed temporal bone computer tomography (CT) and internal auditory canal magnetic resonance imaging (IAC MRI) for all participants to identify any anomalies. A diagnosis of DFNB4 required both radiologic confirmation of an enlarged vestibular aqueduct (EVA) via temporal bone CT or IAC MRI, and molecular genetic confirmation of *SLC26A4* variants. Patients exhibiting a single heterozygous pathogenic *SLC26A4* variant were also classified under DFNB4 if they presented with radiologic evidence of EVA and no indications of branchio-oto-renal syndrome.

This study received approval from the Institutional Review Board of Seoul National University Bundang Hospital (IRB No: B-2204-750-103) and adhered to the principles of the Declaration of Helsinki.

### Audiologic evaluation

All participants underwent comprehensive audiological assessments prior to surgery, which included pure tone audiometry, various speech evaluation tests, and SIR [3–7]. To evaluate the outcomes of cochlear implantation, we analyzed speech evaluation tests conducted at least three months postoperatively. This analysis focused on the correct percentage of sentence perception tests of their last visit as described in recent literature (Korean version of the Central Institute of Deafness) [8–12]. Given the inclusion of patients with extended durations of deafness, who often face significant challenges in articulation and pronunciation, this study also incorporated the SIR as part of the preoperative evaluation. While SIR is traditionally used for assessing postoperative performance in pediatric CI patients, its application here aimed to gauge the spoken language status of our patients. The specific evaluation criteria utilized were derived from Table 1 in the study by Allen et al. [3].

**Table 1:** Subjects’ sentence percent-correct score of their last visit data

Age group	Sentence score (mean ± SD, %)
Total	80 ± 31.0
10 s	91.3 ± 19.4
20 s	76.1 ± 37.2
30 s	83.4 ± 28.1

### Molecular genetic analysis

Molecular genetic testing was conducted for etiologic diagnosis of hearing loss whenever possible. Genomic DNA samples were extracted from the buccal mucosa cells or peripheral blood via standard procedures. Real-time polymerase chain reaction (RT-PCR; U-TOP™ HL Genotyping Kit, SeaSun Biomaterials, Daejeon, Korea) [13] and whole-exome sequencing (WES; Otogenetics, Norcross, GA, USA) was performed. RT-PCR was for the screening of 11 variants of 5 genes (*GJB2*, *SLC26A4*, *MTRNR1*, *TMPRSS3*, and *CDH23*) showing high prevalence in Koreans. Whole-exome sequences were captured by the NimbleGen Sequence Catcher (Roche NimbleGen Inc., Madison, WI, USA) and a SureSelect 50 Mb Hybridization and Capture kit. Bioinformatics analyses were performed as described previously against targeted genes related to hearing loss [14–17]. In total, 32–69 million short reads (100-bp paired-end reads) were obtained via WES. More than 85% of the target exon regions were covered by at least 5 sequence reads. The reads were mapped onto the UCSC hg19 reference genome. Non-synonymous single nucleotide polymorphisms (SNPs) were filtered at a depth ≥ 15. The minor allele frequency (MAF) of the variants was evaluated from publicly available databases: ExAC (http://exac.broadinstitute.org/), 1000 Genomes (https://www.ncbi.nlm.nih.gov/variation/tools/1000genome), TOPMED (https://www.nhlbiwgs.org/), and GnomAD (http://gnomad.broadinstitute.org/)17. Variants with MAF ≥ 0.5% were excluded unless they were previously reported to be pathogenic in the literature, ClinVar, or DVD (http://deafnessvariationdatabase.org/). We used a threshold of 0.5% for autosomal recessive nonsyndromic hearing loss and 0.05% for autosomal dominant nonsyndromic hearing loss.18 Finally, the scores of SIFT (http://sift.jcvi.org/), PolyPhen2 (http://genetics.bwh.harvard.edu/pph2/), GERP (http://mendel.stanford.edu/SidowLab/downloads/gerp/), and CLINVAR (https://www.ncbi.nlm.nih.gov/clinvar/) were used to assess the pathogenicity of candidates.

### Statistical analysis

All statistical analyses were performed using SPSS for Windows version 25 (IBM Corporation, Armonk, NY, USA). Data were visualized using the GraphPad Prism 7.00 (GraphPad Software, California, United States). Data are presented as means ± standard deviation (SD).

## Results

### Demographic and clinical characteristics

**Fig. 1:**
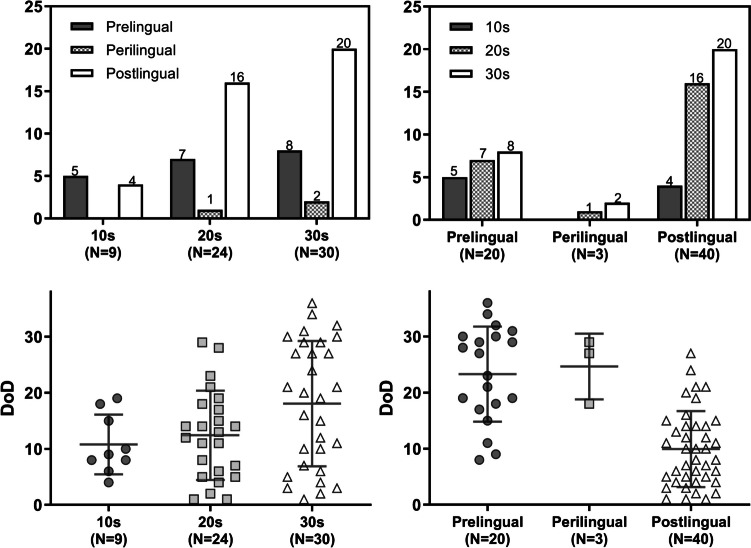
The number of patients and the duration of deafness according to the age at cochlear implantation and the onset of hearing loss. DoD: duration of deafness

Of the 421 subjects, 100 (23.6%) were aged between 10 and 39 years at the time of cochlear implantation. Within this group, 63 subjects underwent their first cochlear implant surgery between the ages of 10–39, while the remaining 37 underwent either a second sequential surgery or revision surgery within this age range. The participant group displayed a female majority: 24 males (38.1%) and 39 females (61.9%). Over half of the subjects (40, 63.5%) experienced postlingual-onset deafness (Fig. 1). The DoD was significantly longer in individuals with prelingual and perilingual hearing loss compared to those with postlingual hearing loss (23.3 ± 8.5, 24.7 ± 5.9, and 9.9 ± 6.8 years, respectively; p < 0.0001, = 0.0033, respectively).

### Etiology of hearing loss

**Fig. 2:**
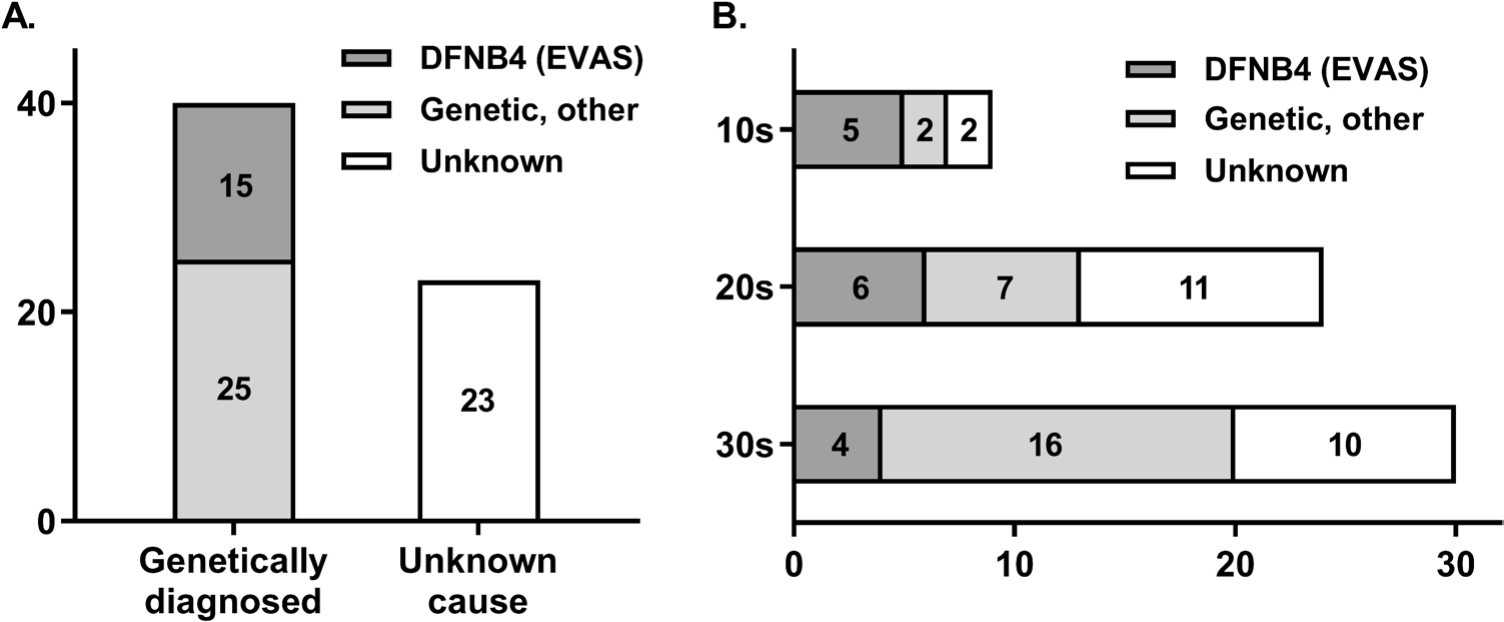
The number of patients according to etiology of hearing loss. **A** Classification by genetic diagnosis. **B** Classification by age group at the time of cochlear implantation

Among the 63 subjects, 61 underwent molecular genetic testing, and 40 (65.2%) were genetically diagnosed (Fig. 2). DFNB4 (Enlarged vestibular aqueduct syndrome, EVAS) was the most common molecular etiology in cochlear implantees aged 10–39, accounting for 15 (37.5%) of the 40 genetically diagnosed cases. The identified *SLC26A4* variants in this cohort included p.H723R, c.919-2A > G, p.T410M, p.Q421P, c.1149 + 3A > G, p.S28R, and c.1001 + 168_c.1545-924del.

Other molecular etiologies in this cohort included variants in *LOXHD1*, *GJB2*, *USH2A,* and other genes. One patient, not genetically tested, was diagnosed with Neurofibromatosis type 2 based on familial history and radiological evidence. Further details are provided in Table S1. The clinical course of implantees due to major other genes (*LOXHD1* and *GJB2*) has already been published by our group [18, 19].

**Fig. 3:**
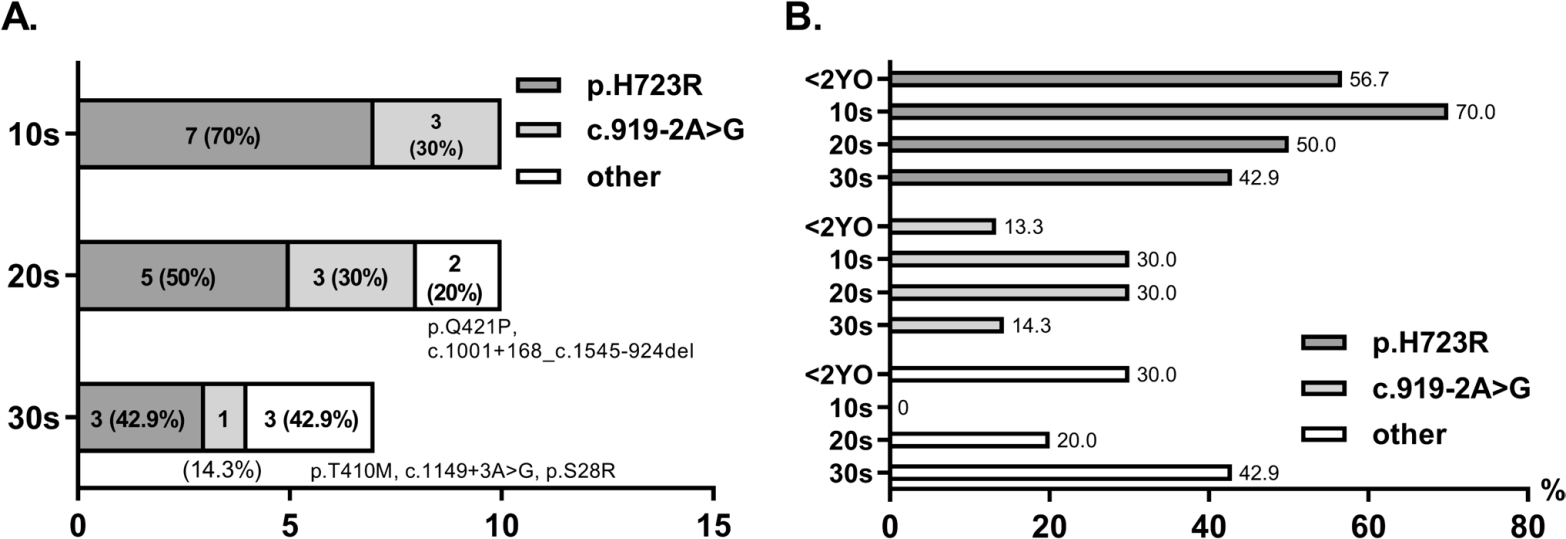
Genotypes of *SLC26A4* variants in DFNB4 subjects. **A** Number of patients by age group and genotype. **B** Variant detection rates in each age group

The prevalence of DFNB4 in our cohort decreased with increasing age, from 55.6% in teens to 25% and 13.3% in the 20 s and 30 s, respectively. Conversely, hearing loss due to other genetic causes without anatomical abnormalities increased, representing 22.2%, 29.2%, and 53.3% in their teens, 20 s, and 30 s, respectively. This suggests that Mendelian genetic hearing loss is not solely a pediatric issue. In the case of DFNB4, the most common molecular etiology, *SLC26A4* variants were analyzed by genotype (Fig. 3). In alignment with known patterns in East Asian populations, the most prevalent variant was p.H723R, followed by c.919-2A > G. These two variants were the only ones identified in teenagers, with a more diverse range of variants observed in older age groups: 20% in their twenties and 42.9% in their thirties (Fig. 3A). We also compared this data with the genotype data of 16 DFNB4 patients who received CI at our hospital before the age of two years to observe variant genotype changes with age (Fig. 3B). Among these 16 children, 30 mutant DFNB4 alleles were identified, with the proportion of c.919-2A > G differing significantly between early (< 2 years old) and late (teens to 30 s) DFNB4 implantees. Specifically, only 13.3% (4/30) among the early implantees had c.919-2A > G, while it nearly doubled to 25.9% (7/27) among the late implantees.

## Outcomes of cochlear implantation

**Fig. 4:**
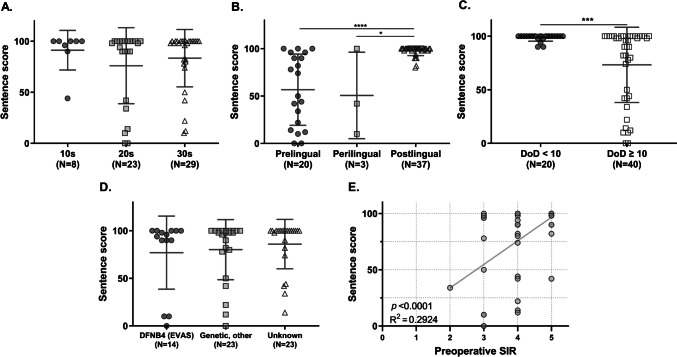
Sentence score by the group based on age at CI by decade, onset of hearing loss, duration of deafness, etiology of hearing loss, and simple linear analysis with preoperative speech intelligibility rating. DoD: duration of deafness (years); SIR: speech intelligibility rating

To assess the speech recognition improvement post-CI, 60 subjects underwent speech evaluation tests at least three months following surgery. The analysis focused on the sentence percent-correct scores from their last visit (Table 1, Fig. 4). The average follow-up period was 17.2 months (range 3-50 months, SD = 10.0). The average sentence score for these 60 subjects was 80% (SD = 31.0). Notably, scores varied by age group: teens averaged 91.3 ± 19.4, those in their 20 s averaged 76.1 ± 37.2, and those in their 30 s averaged 83.4 ± 28.1.

Subjects were categorized based on five variables: decade age at the time of CI, onset of hearing loss, DoD, etiology of hearing loss, and preoperative SIR, to identify potential prognostic factors influencing CI outcomes. The average sentence scores for each group were compared. Age categorization did not show a significant difference in sentence scores (*p* = 0.5388, Kruskal–Wallis test) (Fig. 4A); however, a bimodal distribution was observed in subjects in their 20 s, with six scoring 42 or lower and 17 scoring 90 or higher, including 10 with perfect scores. No other features were identified among the six low-scoring participants.

Grouping by the onset of hearing loss, postlingual cases showed superior performance compared to pre-lingual and peri-lingual groups (Fig. 4B, p < 0.0001, 0.0359 respectively, Mann–Whitney test). The cohort was further based on DoD (< 10 years vs. ≥ 10 years). Significant differences were observed in sentence scores, with the shorter DoD group outperforming the longer DoD group (Fig. 4C, p = 0.0007, Mann–Whitney test). Notably, in post-lingual cases, favorable outcomes were observed even with a DoD exceeding 10 years (n = 18, 97 ± 6.3) **(**Fig. 4B**)**. However, genetic etiology did not significantly influence speech recognition performance (*p* = 0.2529, Kruskal–Wallis test) (Fig. 4D). Univariate simple regression analysis revealed a positive correlation between preoperative SIR and post-CI sentence scores (*p* < 0.0001, Fig. 4E).

A univariate linear regression analysis was conducted to assess the impact of these five variables on postoperative sentence scores. Initial univariable simple linear regression analysis identified significant correlations with three variables: onset of hearing loss, DoD, and preoperative SIR (Table 2). Subsequent multiple linear regression analysis, including only these significant variables, confirmed that both the onset of hearing loss and SIR significantly affected sentence scores. The variance inflation factors (VIF) in this analysis were all below 5, indicating no multicollinearity among these variables.

**Table 2:** Univariate simple and multiple linear regression of factors associated with sentence score after cochlear implantation

Factors	Simple LR analysis		Multiple LR analysis		
	Coefficient (95% CI)	*p*-value	Coefficient (95% CI)	*p*-value	VIF
Age by decade at CI	− 0.843 (− 12.343, 10.656)	0.884	–		
Onset of hearing loss	20.920 (14.217, 27.623)	0.000	16.666 (8.543, 24.789)	0.000	1.570
Duration of deafness	− 21.905 (− 37.849, -5.961)	0.008	− 4.463 (− 18.286, 9.361)	0.520	1.217
Etiology of hearing loss	4.628 (− 5.790, 15.047)	0.378	–		
Preoperative SIR	20.932 (12.195, 29.569)	0.000	9.393 (0.303, 18.482)	0.043	1.466

LR: linear regression; SIR: speech intelligibility rating; VIF: variance inflation factor

## Discussion

As of June 2021, teenagers comprised 9.2% of South Korea’s population, with those in their twenties and thirties each accounting for 13.1% of the total population. Collectively, this age group (10 s to 30 s) represented 35.3% of the population [20]. However, adolescents and young adults form the smallest proportion of the overall CI population, as noted in literature [21]. Our study similarly found that the percentage of CI recipients in their teens to thirties (15.0%) was significantly lower than their overall population proportion (35.3%) in South Korea. This discrepancy likely contributes to the limited research focus on CI in adolescents and young adults.

In fact, earlier research focused solely on adolescents and young adults with pre-lingual hearing loss. CI has been reported to be advantageous to them, although the results were variable, and their performance was poorer than that of those with post-lingual deafness [22–24]. Moreover, preoperative hearing aid use and postoperative education were known to have a positive influence on the outcomes [23, 25]. In a self-report questionnaire study, late-implanted adults reported feeling more satisfied with their lives after CI than before [26]. It has been found that individuals who underwent CI during adolescence have higher rates of non- and partial use than their adolescent counterparts who were implanted during childhood [27]. Besides, age at implantation and interimplant interval were not correlated with performance parameters in a study of sequential bilateral CI in adolescents [28]

Diverging from these studies, our current research included adolescent and young adult cochlear implantees with various onsets of hearing loss. When compared to the actual age-specific population distribution, the proportion of implantees in their 20 s to 30 s (12.8%) was notably lower than that in the general population (26.2%), while the proportion of teenage implantees (2.14%) was also significantly lower than their counterpart in the general population (9.2%). This suggests a decline in teenage implantees, likely due to early CI in cases of early-onset hearing loss. The inclusion of a significant number of CI patients in their 20 s or 30 s indicates the likelihood of long DoD, spanning 20 to 30 years, in cases of both prelingual and postlingual hearing loss. This diversity allowed for a more comprehensive analysis of CI outcomes using various variables. Our study focuses on a unique group outside the categories, potentially with prelingual deafness or extended sound deprivation. Investigating postoperative outcomes and prognostic factors in this group was crucial.

In a previous study examining postlingually deaf CI recipients with genetic diagnoses, the K-CID score averaged 88.8 with a standard deviation of 5.0 [11]. In contrast, our study reported an average K-CID score of 80 with a larger standard deviation of 31.0, indicating a broader range of performance levels. This underscores the importance of identifying predictive factors for auditory performance in our patient group.

Our univariable simple regression analysis identified DoD, onset of hearing loss (prelingual, perilingual vs. postlingual), and SIR as significant contributors to post-CI speech outcomes. In the multiple linear regression analysis, however, DoD lost its significance. While the obtained VIFs suggest addressing multicollinearity, it's plausible that DoD was highly correlated with either onset of hearing loss or preoperative SIR. Alternatively, the non-significant results in this analysis might be attributable to sample size limitations. Accurately determining the onset of severe hearing loss can be challenging in clinical settings. Genetic testing, while helpful in identifying the molecular etiology and known audiologic phenotype, often leaves many cases with elusive onset times. In such instances, SIR, which is closely associated with prognosis, becomes a valuable predictor of CI outcomes in this age group.

Previous studies predominantly utilized SIR as an outcome metric post-CI, with pediatric subjects showing a gradual post-surgery increase [29–31]. Studies involving congenitally deaf adults also found that a favorable preoperative SIR is associated with improved post-CI performance [32, 33]. Our research extends these findings by including individuals with diverse onset profiles of hearing loss, ranging from teens to thirties. Thus, SIR emerges as an important indicator and potential predictor for future speech outcomes in our unique study population. Given its simplicity, SIR could be more widely employed in clinical settings.

Genetic diagnosis was feasible in approximately two-thirds of patients undergoing CI between ages 10 and 39, with about one-fourth having EVA. The presence or absence of a genetic diagnosis or specific gene variants did not significantly influence surgical outcome predictions in this study. This may be attributed to the variety of causative genes in this age group and their diverse impacts on CI outcomes. However, knowledge of these genes can assist in estimating onset age or DoD and provide evidence for recommending CI surgery in patients with favorable SIR scores [34, 35]. According to Lee et al. (2020), patients with postlingual hearing loss fared better when timely CI surgery was performed after a mutation in a known hearing loss gene was discovered [11]. Additionally, this study highlights the SLC26A4 c.919-2A > G variant, prevalent in a significant portion of our patient cohort in their teens and twenties. While many patients with this variant and diagnosed with DFNB4 typically undergo surgery before age 10 due to prelingual or perilingual hearing loss, our findings indicate that a considerable number also present with postlingual hearing loss. It is important to note that the c.919-2A > G variant is generally associated with a milder phenotype compared to the p.H723R mutation at least in Koreans [36, 37], although this observation has not been replicated in neighboring populations such as Mongolians and Japanese yet [38, 39].

DoD also influenced CI outcomes in this age demographic. Consistent with previous studies [40], we found that CI outcomes were poorer with DoD exceeding 10 years (Fig. 4C). However, in individuals with postlingual hearing loss, DoD did not impact their post-CI performance; results were similar regardless of whether DoD exceeded 10 years or was less than 10 years (Fig. 4B). This finding is significant as the DoD within our study's target age range is often extended, suggesting the feasibility of CI irrespective of DoD in cases of postlingual hearing loss.

In acknowledging the limitations of this study, several factors warrant consideration. First, the retrospective design of our investigation inherently introduces potential biases, including those associated with data collection and analysis. Given that our study relies on pre-existing medical records, there is an unavoidable risk of incomplete data and the possibility of recall bias affecting the reported outcomes. Additionally, the focus on a specific demographic group—adolescents and young adults aged 10 to 39 years—and the inclusion of subjects operated on by a single surgeon at a single center may limit the generalizability of our findings. This demographic and clinical setting might not accurately represent broader populations across different healthcare environments. Furthermore, the relatively small sample size of 63 participants could constrain our ability to detect subtle effects or associations, particularly when analyzing stratified subgroups by age or genetic etiology. These limitations suggest the need for caution in extrapolating our results too broadly and underscore the importance of validating our findings through larger, multi-center prospective studies.

## Conclusion

Our study underscores the underrepresentation of adolescents and young adults (ages 10 to 30) in CI populations, a finding that stands in contrast to their substantial proportion in the general population. This research highlights that the onset timing of hearing loss and preoperative SIR are pivotal predictors of postoperative outcomes in this demographic. For individuals in this age group with postlingual hearing loss, CI is recommended regardless of the DoD. Furthermore, the study accentuates the significance of genetic factors in the etiology of hearing loss among implantees in their teens, twenties, and thirties, and reinforces the predictive value of preoperative SIR for CI outcomes.

## Supplementary Information

Below is the link to the electronic supplementary material.

Supplementary file 1 (DOCX 22 KB)

## Data Availability

The datasets generated during and/or analyzed during the current study are available from the corresponding author on reasonable request.
