# Supplementary material for: Clinical characteristics and hearing loss etiology of cochlear implantees undergoing surgery in their teens, 20s, and 30s
Source: Eur Arch Otorhinolaryngol. 2024 May 27;281(10):5169–77. doi: 10.1007/s00405-024-08737-3 (PMC11416388; doi:10.1007/s00405-024-08737-3)
Supplement: Supplementary file 1 — (DOCX 22 KB) [file 405_2024_8737_MOESM1_ESM.docx]

**Supplementary Table 1**.

| **Case No.** | **Sex** | **Age** | **Side** | **Gene** | **Mutation** | **Reported onset pattern in the literature** | **Patient’s onset in this study** |
| --- | --- | --- | --- | --- | --- | --- | --- |
| 1 | F | 28 | R | *Mitochondrial* | Mitochondrial 3243 | Post | Post |
| 2 | M | 28 | R | *GJB2* | c.299delAT (heterozygote), c.235delC (heterozygote) | Pre | Pre |
| 4 | F | 34 | R | *LOXHD1* | c.4212+1G>A (homozygote) | Pre, Post | Post |
| 6 | M | 21 | L | *SLC26A4* | p.H723R (heterozygote), c.1001+168_c.1545-924del (heterozygote) | Pre, Post | Pre |
| 7 | M | 25 | L | *SLC26A4* | p.His723Arg (heterozygote), c.919-2A>G (heterozygote) | Pre, Post | Pre |
| 8 | F | 31 | R | *SLC26A4* | p.H723R (heterozygote), c.1149+3A>G (heterozygote) | Pre, Post | Post |
| 9 | M | 36 | L | *Mitochondrial* | Mitochondrial | Post | Post |
| 10 | M | 26 | B | *SLC26A4* | c.919-2A>G (heterozygote) | Pre, Post | Pre |
| 11 | F | 35 | L | *USH2A* | c.14835delT:p.Ser4945fs (heterozygote), c.13112_13115delAAAT:p.Gln4371fs(heterozygote) | Pre | Post |
| 12 | F | 32 | L | *MYO15A* | p.R1835H (heterozygote), p.S3417del (heterozygote) | Pre, Post | Post |
| 13 | F | 39 | B | *GJB2* | c.235delC (homozygote) | Pre | Pre |
| 15 | F | 34 | B | *SLC26A4* | p.T410M (heterozygote), p.H723R (heterozygote) | Pre, Post | Peri |
| 17 | F | 25 | B | *SLC26A4* | c.919-2A>G (heterozygote) | Pre, Post | Post |
| 20 | F | 33 | R | *LOXHD1* | p.Ser1534fs (heterozygote), c.4212+ 1G>A (heterozygote) | Pre, Post | Pre |
| 22 | M | 18 | R | *SLC26A4* | c.2168A>G:p.H723R (homozygote) | Pre, Post | Pre |
| 24 | M | 26 | B | *MYO7A* | c.1133G>A:p.Arg378His (heterozygote), c.5186C>T:p.Thr1729Met (heterozygote) | Pre, Post | Post |
| 25 | F | 32 | R | *CDH23* | c.C719T:p.P240L (heterozygote), c.C4762T:p.R1588W (heterozygote) | Pre, Post | Post |
| 26 | F | 28 | L | *DFNA5* | c.991-15_991-13delTTC (heterozygote) | Post | Post |
| 28 | F | 36 | B | *POU4F3* | p.Gly221Glufs*77 (heterozygote) | Post | Post |
| 31 | F | 27 | L | *SLC26A4* | p.H723R (homozygote) | Pre, Post | Post |
| 32 | M | 31 | L | *SLC26A4* | c.919-2A>G (heterozygote) | Pre, Post | Post |
| 33 | F | 31 | L | *LMX1A* | c.622C>T:p.Arg208Ter (heterozygote) | Pre, Post | Post |
| 35 | M | 31 | L | *LOXHD1* | c.4600_4601delTC:p.Ser1534fs (heterozygote), c.4212+1G>A (heterozygote) | Pre, Post | Pre |
| 36 | M | 31 | B | *TMC1* | c.1714G>A:p.Asp572Asn (heterozygote) | Post | Post |
| 37 | F | 24 | R | *NF2* | Diagnosed by familial history and radiologic confirmation | Post | Post |
| 38 | M | 16 | L | *SLC26A4* | c.919-2A>G (heterozygote), p.H723R (heterozygote) | Pre, Post | Post |
| 41 | M | 19 | L | *USH2A* | c.1048G>A:p.Val350Ile (heterozygote), c.8559-2A>G (heterozygote) | Pre | Post |
| 45 | F | 12 | B | *SLC26A4* | c.919-2A>G (heterozygote), p.H723R (heterozygote) | Pre, Post | Pre |
| 47 | M | 24 | L | *SLC26A4* | p.H723R (heterozygote), p.Q421P (heterozygote) | Pre, Post | Post |
| 48 | F | 38 | B | *SLC26A4* | p.H723R (heterozygote), p.S28R (heterozygote) | Pre, Post | Peri |
| 49 | M | 15 | L | *POU3F4* | c.499C>T:p.Arg167Ter (hemizygote) | Pre | Pre |
| 50 | F | 14 | L | *SLC26A4* | c.919-2A>G (heterozygote), p.H723R (heterozygote) | Pre, Post | Post |
| 53 | M | 21 | L | *MYH9* | c.2107A>T:p.Ile703Phe (heterozygote) | Post | Post |
| 54 | F | 27 | R | *CDH23* | c.1205C>T:p.Pro402Leu (heterozygote), c.5138C>A:p.Ala1713Asp (heterozygote) | Pre | Post |
| 56 | M | 19 | B | *SLC26A4* | p.H723R (homozygote) | Pre, Post | Pre |
| 57 | F | 31 | B | *COL11A2* | c.966dup:p.Thr323Hisfs*19 (heterozygote), c.4294G>T:p.Gly1432* (heterozygote) | Post | Post |
| 58 | F | 34 | B | *PTPN11* | c.5C>T:p.Thr2Ile (heterozygote) | Pre | Pre |
| 59 | F | 34 | B | *PTPN11* | c.5C>T:p.Thr2Ile (heterozygote) | Pre | Pre |
| 61 | M | 35 | B | *TMPRSS3* | c.916G>A:p.Ala306Thr (homozygote) | Pre, Post | Post |
| 63 | M | 30 | L | *GJB2* | c.235del:p.Leu79Cysfs*3 (homozygote) | Pre | Pre |

Pre, prelingual; Peri, perilingual; Post, postlingual.
